# CpG DNA methylation changes during epididymal sperm maturation in bulls

**DOI:** 10.1186/s13072-023-00495-6

**Published:** 2023-05-30

**Authors:** Emanuele Capra, F. Turri, B. Lazzari, S. Biffani, A. Lange Consiglio, P. Ajmone Marsan, A. Stella, F. Pizzi

**Affiliations:** 1 Institute of Agricultural Biology and Biotechnology, National Research Council IBBA CNR, 26900 Lodi, Italy; 2 Department of Veterinary Medicine and Animal Sciences (DIVAS), Università degli Studi di Milano, 26900 Lodi, Italy; 3 Department of Animal Science, Food and Technology-DIANA, and Romeo and Enrica Invernizzi Research Center on Sustainable Dairy Production-CREI, Università Cattolica del Sacro Cuore, 29122 Piacenza, Italy; 4 Institute of Agricultural Biology and Biotechnology, National Research Council IBBA CNR, 20133 Milano, Italy

**Keywords:** Sperm, Epididymis, Methylation, Bull, Caput, Cauda, Corpus, Motility, Sperm kinetics, Chromatin integrity

## Abstract

**Background:**

During epididymal transit spermatozoa acquire specific morphological features which enhance their ability to swim in a progressive manner and interact with the oocytes. At the same time, sperm cells undergo specific molecular rearrangements essential for the fertilizing sperm to drive a correct embryo development. To assess epigenetic sperm changes during epididymal maturation, the caput, corpus and cauda epididymis sperm tracts were isolated from eight bulls and characterized for different sperm quality parameters and for CpG DNA methylation using Reduced Representation Bisulfite Sequencing (RRBS) able to identify differentially methylated regions (DMRs) in higher CpG density regions.

**Results:**

Caput sperm showed significant variation in motility and sperm kinetics variables, whereas spermatozoa collected from the corpus presented morphology variation and significant alterations in variables related to acrosome integrity. A total of 57,583 methylated regions were identified across the eight bulls, showing a significantly diverse distribution for sperm collected in the three epididymal regions. Differential methylation was observed between caput vs corpus (*n* = 11,434), corpus vs cauda (*n* = 12,372) and caput vs cauda (*n* = 2790). During epididymal transit a high proportion of the epigenome was remodeled, showing several regions in which methylation decreases from caput to corpus and increases from corpus to cauda.

**Conclusions:**

Specific CpG DNA methylation changes in sperm isolated from the caput, corpus, and cauda epididymis tracts are likely to refine the sperm epigenome during sperm maturation, potentially impacting sperm fertilization ability and spatial organization of the genome during early embryo development.

**Supplementary Information:**

The online version contains supplementary material available at 10.1186/s13072-023-00495-6.

## Background

Spermatozoa are highly specialized cells which possess unique features. Compared to other cells, they use a flagellum for movement and possess a distinctive DNA structure and chromatin packaging. These unique features are the results of morphogenetic events during spermiogenesis in the testis and during sperm maturation in the epididymal transit [1]. Although defective spermatogenesis is the most well-known cause of sperm dysfunctions, inadequate epididymal maturation deeply affects sperm quality and may influence male fertility [2, 3].

The mammalian epididymis is a long convoluted ductal system divided into different regions. It supports sperm maturation through a regionalized functionality, triggered by the interaction within the surrounding epithelium [4]. In humans, epididymis presents a three-dimensional structure of about 10 cm in length, containing a 6 m long duct packed in a well-organized structure [5]. In other species, the epididymal duct is variable in size: about 1 m in mice [6], from 40 to 60 m in cattle [7], and 80 m in horses [8]. Epididymis can be divided into four regions: the initial segment, caput, corpus and cauda, with unique characteristics and that differently contribute to sperm maturation [9]. Each segment is specialized in synthetizing and releasing specific molecules that are prevalently channeled through extracellular vesicles named epididymosomes. Epididymosomes can directly promote sperm maturation and alter sperm composition by delivering regulatory cargo molecules such as proteins [10], small non-coding RNAs (sncRNA) [11, 12] and lipids [13]. Different classes of sncRNA were observed to be specifically conveyed in different tracts of the epididymis, changing sperm RNA payload [14, 15]. During epididymal transit, spermatozoa undergo a remodeling of the epigenomic signature, with changes both in the ncRNAs cargo and in chromatin organization, essential for promoting a correct sperm maturation, embryo development, and modulating epigenetic inheritance [16]. Sperm isolates from different tracts of the epididymis show a remarkable level of plasticity in term of ncRNAs expression [17] and chromatin histone post-translational modification [18]. During epididymis transit, sperm epigenetic remodeling is accompanied by profound changes in sperm concentration, morphology, motility, permeability, chromatin condensation and metabolism, necessary to obtain a correct maturation and functionality of the sperm [19].

Flow cytometry (FCM) is an excellent tool for the evaluation of semen characteristics [20]. It allows to analyze many thousand sperms within a short time, obtaining an accurate and unbiased evaluation of their integrity and functionality. Among the various physiological features, a good evaluation of sperm DNA damage, chromatin structure alterations extrapolated from the sperm chromatin structure assay (SCSA) and viability and acrosome integrity give a good indication of the quality of the maturation of epididymal sperm cells. The measure of chromatin compaction and sperm head morphology were found to be linked to sperm epididymal maturation and to be potentially useful in predicting fertility in bulls [21, 22]. In addition, sperm chromatin was also observed to be different in young versus adult bulls [23]. Sperm from young bulls presents a higher motility, plasma and acrosomal membrane integrity, and high mitochondrial potential, but also defective protamination, causing a higher susceptibility to DNA damage [23].

DNA methylation is essential to facilitate the correct chromatin organization and structure [24]. CpG methylation changes were observed in very young, early pubertal bulls suggesting that establishment of DNA methylation patterns in male gametes is a dynamic process that clearly evolves as the animal matures [25]. DNA methylation in specific loci was also observed to increase with age in human [26]. The intra-individual plasticity of the bull sperm methylome can be related to both age and fertility [27]. Recently, sperm methylome profiling of 120 bull ejaculates identified DNA methylation profiles predisposing to subfertility [28].

A full understanding of mechanisms changing DNA methylation during sperm epididymal transit is still lacking. Given the suggested role of DNA methylation in regulating sperm chromatin structure, this study was designed to investigate in parallel the dynamic of semen quality and CpG methylation variation during epididymal sperm maturation. Reduced Representation Bisulfite Sequencing (RRBS) and sperm quality analysis were assessed in bull sperm collected from the caput, corpus and cauda of the epididymis, to investigate the variation in sperm methylation and chromatin condensation during sperm epididymal maturation. Considering that bull aging may influence sperm quality parameters and methylation, two groups of young and adult bulls were included in this study.

## Results

### Computer-assisted semen analysis and flow cytometry analysis

A significant increase of the cells progressive motility (PROG), straight-line velocity (VSL), curvilinear velocity (VCL), average path velocity (VAP), linearity of the curvilinear path (LIN), straightness of the average path (STR), wobble (WOB), amplitude of lateral head displacement (ALH) and beat cross-frequency (BCF) was observed in the cauda tract when compared to the caput and the corpus epididymis. Sperm viability and membrane integrity variables significantly decreased going from the caput epididymidis to the corpus epididymidis, and improved from the corpus to the cauda epididymidis. In the corpus epididymis, significant alterations in variables describing acrosome integrity were also detected, such as in viable sperm with intact acrosome (VI), viable sperm with disrupted acrosome (VR), in sperm death with intact acrosome (DI), in sperm death with disrupted acrosome (DR). Interestingly, in the corpus epididymidis tract, sperm alterations were observed that are potentially related to chromatin structure, together with significant changes in the proportion of immature spermatozoa lacking the normal exchange of histone for protamine-complexed DNA (HDS); Table 1.

**Table 1:** Quality parameters for sperm isolated from caput, corpus, cauda epididymis tracts

Parameter	Epididymal tracts			P-value
	Caput	Corpus	Cauda	
MOT_TOT (%)	61.76 ± 6.03	70.31 ± 6.03	74.25 ± 6.03	NS
STAT (%)	38.23 ± 6.03	29.68 ± 6.03	25.75 ± 6.03	NS
PROG (%)	15.08 ± 2.61a	19.50 ± 2.61a	**39.93 ± 2.61 b**	< 0.001
NOPROG (%)	46.61 ± 5.05	50.86 ± 5.05	34.30 ± 5.05	NS
VCL (µm/s)	89.51 ± 4.27 a	97.59 ± 4.27 a	**120.05 ± 4.27 b**	< 0.0001
VSL (µm/s)	18.48 ± 4.02 a	22.49 ± 4.02 a	**59.81 ± 4.02 b**	< 0.001
VAP (µm/s)	33.70 ± 3.91 a	38.65 ± 3.91 a	**73.15 ± 3.91 b**	< 0.001
LIN (%)	20.69 ± 2.64 a	22.89 ± 2.64 a	48.95 ± 2.64 a	< 0.0001
STR (%)	55.41 ± 2.48 a	57.33 ± 2.48 a	**80.15 ± 2.48 b**	< 0.0001
WOB (%)	37.61 ± 2.62 a	39.52 ± 2.62 a	**60.34 ± 2.62 b**	< 0.0001
ALH (µm)	3.52 ± 0.16 a	3.93 ± 0.16 a	3.97 ± 0.16 a	NS
BCF (Hz)	4.78 ± 0.36 a	5.32 ± 0.36 a	**7.09 ± 0.36 b**	< 0.0001
V (%)	62.02 ± 3.13 a	**53.60 ± 3.13 ab**	**65.87 ± 3.13 ac**	< 0.05
D (%)	37.98 ± 3.15 a	**46.70 ± 3.15 b**	34.16 ± 3.15 a	< 0.05
VI (%)	61.00 ± 3.16 a	**51.13 ± 3.16 b**	64.13 ± 3.16 a	< 0.05
VR (%)	1.01 ± 0.32 a	**2.47 ± 0.32 b**	1.71 ± 0.32 a	< 0.05
DI (%)	33.07 ± 2.58 a	**37.91 ± 2.58 ab**	**27.10 ± 2.58 ac**	< 0.05
DR (%)	4.90 ± 0.79 a	**8.78 ± 0.79 b**	7.06 ± 0.79 a	< 0.05
Alpha-T	0.41 ± 0.001a	**0.40 ± 0.001ab**	**0.40 ± 0.002bc**	< 0.05
ATSD	0.012 ± 0.001	0.016 ± 0.001	0.0153 ± 0.001	NS
DFI (%)	1.003 ± 0.236	1.638 ± 0.236	1.567 ± 0.253	NS
HG (%)	**1.527 ± 0.435a**	**2.115 ± 0.435ab**	**0.699 ± 0.466ac**	NS
HDS	3.346 ± 0.0449a	**3.531 ± 0.0449b**	3.307 ± 0.0481a	< 0.001

*MOT TOT* total motility, *STAT* immotile cells, *PROG* cells progressive motility, *VSL* straight-line velocity, *VCL* curvilinear velocity, *VAP* average path velocity, *LIN* linear coefficient, *STR* straightness coefficient, *WOB* wobble coefficient, *ALH* amplitude of lateral head displacement, *BCF* beat cross-frequency, *V* viable sperm, *D* death sperm, *VI* viable with intact acrosome, *VR* viable with disrupted acrosome, *DI* death with intact acrosome, *DR* death with disrupted acrosome, *Alpha-T* red/(red + green) fluorescence intensity, *ATSD* Alpha-T standard deviation, *DFI* fragmented DNA sperm, *HG* high green fluorescence sperm, *HDS*: proportion of immature spermatozoa lacking the normal exchange of histone for protamine-complexed DNA. a,b,c, values within a row with different superscript letters differ significantly at P < 0.05

Within the three epididymis tracts, sperm quality parameters varied also between young and adult bulls. In particular, older animals showed better values of parameters related to motility and kinetics (PROG, VLC, VSL, VAP, LIN, STR, V, VI), and a lower percentage of dead sperms in sperm isolated from caudal epididymis. Values of the V and VI variables were significantly lower in sperm collected from the caput tract of young bulls compared to old bulls. Conversely, in the same tract M and MI values were significantly higher in young bulls. WOB, ALH and DFI values were significantly lower in sperm collected from the corpus tracts from young bulls, compared to old bulls group (Additional file 1).

### Fluorescence microscope assessment of sperm acrosome membrane

**Fig. 1:**
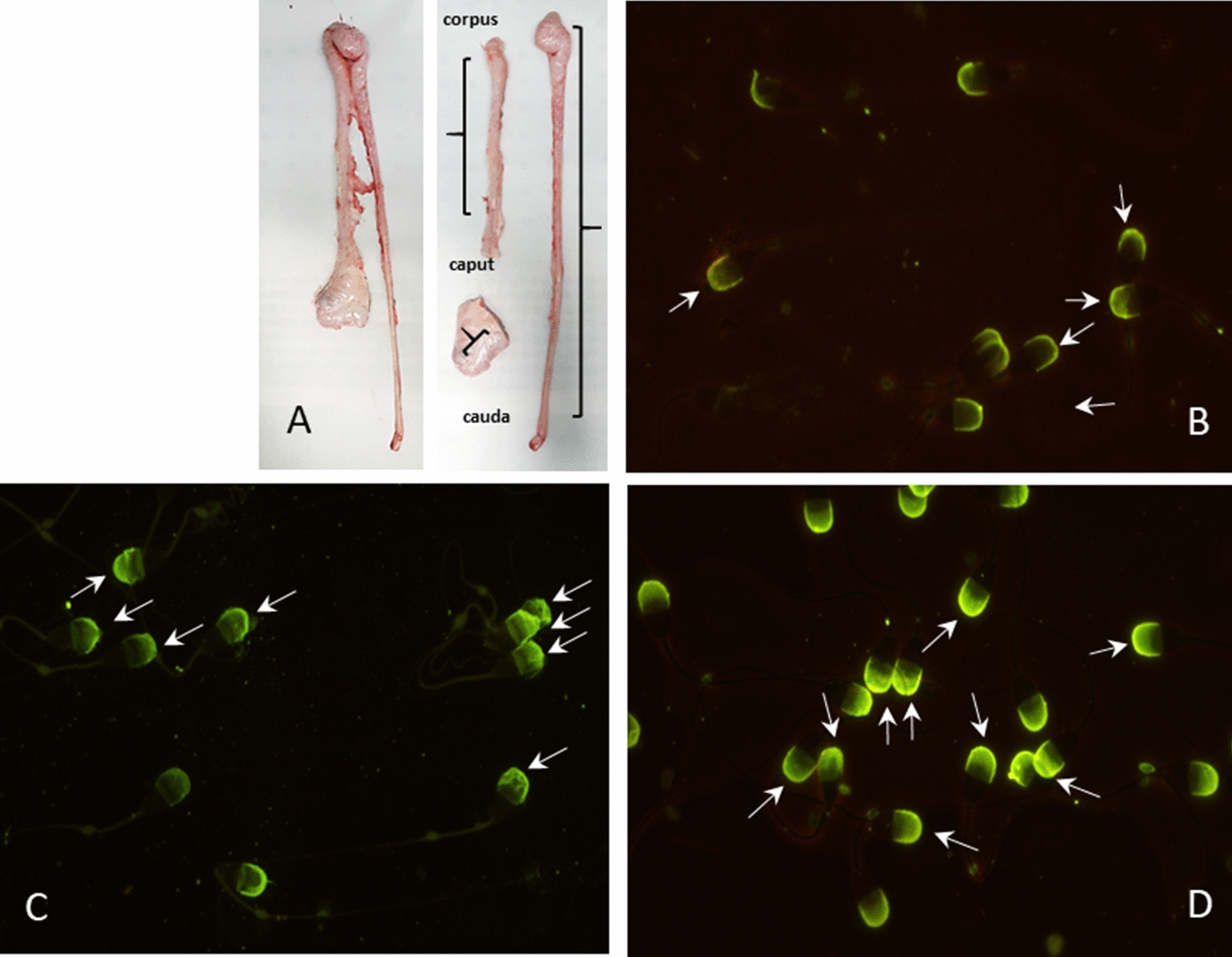
**A** Dissection of epididymis and detection of acrosome in the three epididymis tracts. On the left, epididymis with respective ductus deferens dissected from the testicle. On the right the three epididymis tracts (caput, corpus and cauda with ductus deferens) surgically separated, well distinct from an anatomical point of view and not adjacent. Curly brackets indicate the effective tracts from which sperm cells were recovered. **B**–**D** Sperm acrosomes stained with FITC-PNA. Different patterns of acrosome staining are visible in the three epididymis tracts: **B** slightly disordered membrane acrosome; **C** severely disordered membrane acrosome; **D** intact acrosome with normal membrane

Sperm isolated from three epididymal tracts (Fig. 1A) were also stained with FITC-PNA in order to visually detect structural modifications in sperm head membranes by fluorescence microscopy, through the three different epididymis tracts. From this evaluation the staining patterns of the acrosomes were classified into three categories: slightly disordered acrosome (SLA), severely disordered acrosome (SEA) and intact acrosome (IA) with normal membrane (Fig. 1B–D). Between the different tracts the higher percentage of SEA and SLA and the lower percentage of IA were detected in the corpus tract (SEA, caput: 4,41%; corpus: 9,49; cauda: 3,49%; SLA, caput: 13,51%; corpus: 31,77; cauda: 6,80%; IA, caput: 82,09%; corpus: 58,74; cauda: 89,71%) confirming that the greatest global damage (~ 40%), in terms of sperm acrosome membrane, was found in the corpus tract, against a minor damage in the caput and cauda tract (~ 20% and ~ 10%, respectively).

### Global DNA methylation, principal component and hierarchical clustering analysis

**Fig. 2:**
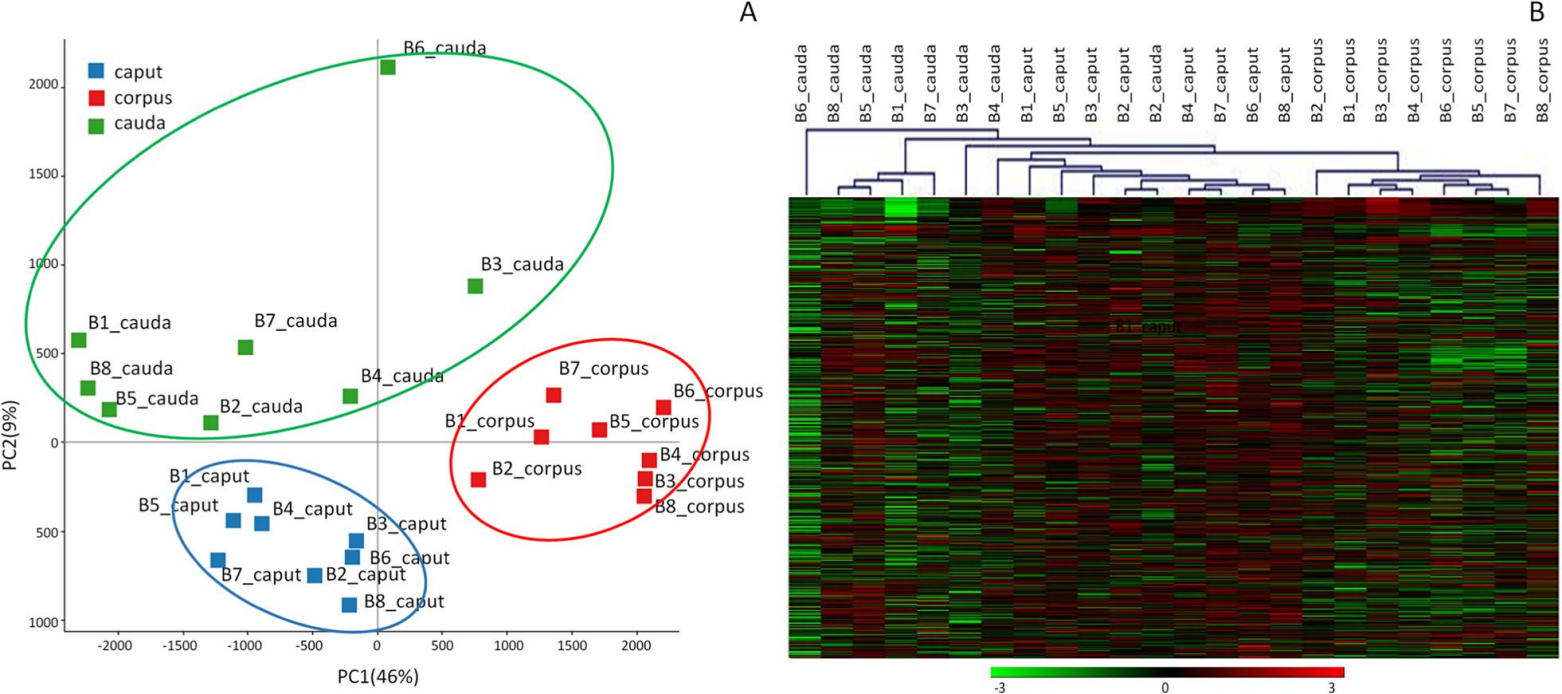
**A** Principal component analysis and **B** hierarchical clustering for caput, corpus and cauda sperms isolated from eight bulls (B1–B8)

Epigenetic variation was evaluated in three epididymis tracts (caput, corpus and cauda) of sperm isolated from 8 bulls (B1-B4 young bulls 16–19 months of age; B5–B8 adult bulls 45–72 month old) by RBBS. An average of 38M reads, a mapping efficiency of about 60% and an average CpG methylation of about 72% were obtained (Additional file 2 for statistics). After applying a 10X coverage cutoff for all cytosines mapped in all animals, a total of 57,583 methylated regions (MRs) of 400 bp in size containing at least 3 close cytosines were identified. Principal component analysis of the total MRs discriminates well sperm isolated from the different epididymis tracts. Conversely, no clustering of the 8 bulls according to age is observed (Fig. 2A).

PC1, which explains 46% of the variance, clearly separates caput and corpus samples, whereas cauda sperms have a less homogeneous distribution and are separated by PC2 that explains 9% of the total variance. Hierarchical clustering shows that sperm isolated from the same epididymis tract group together, separating caput, corpus and cauda samples (Fig. 2B).

### Analysis of differentially methylated regions

Differentially methylated regions (DMRs) in sperm were assessed between young and old bulls and among caput, corpus and cauda epididymis. Sperm showed a modest variation in CpG methylation between young and old bulls, with 379, 1167 and 9 DMRs in the caput, corpus and cauda, respectively (Additional file 3).

**Fig. 3:**
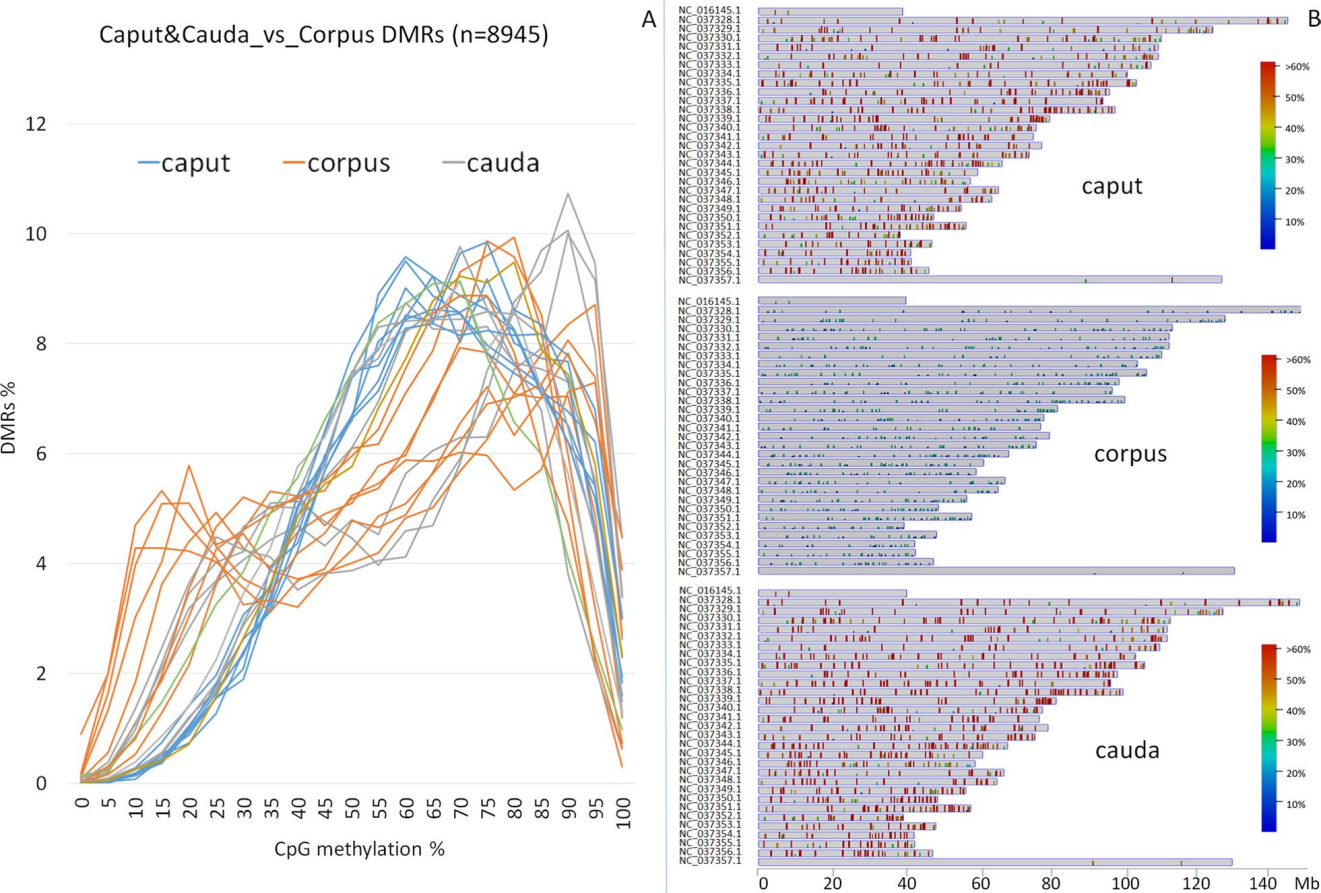
**A** Distribution of methylation in 8945 DMRs shared by the comparisons “caput vs corpus” and “caput vs cauda” in sperm isolated from eight bulls and stratified on the basis of the average methylation level of CpGs (ranging from 0 to 100%). **B** Distribution along chromosomes in caput, corpus and cauda of DMRs with average methylation below 30% in corpus sperm

Sperm isolated from the three different epididymal tracts showed the highest methylation difference, regardless of the age (Fig. 2). A very high number of DMRs was identified: caput vs corpus (DMRs = 11,434), corpus vs cauda (DMRs = 12,372) and caput vs cauda (DMRs = 2790) (Additional file 4). A large number of DMRs (*n* = 8945) was shared by “corpus vs caput” and “corpus vs cauda” comparisons (Additional file 5), most of which were hypomethylated in sperm from the corpus. In these regions methylation profiles of sperm from caput and from cauda were rather similar, while corpus showed distinct methylation patterns (Fig. 3A). A focus on individual regions hypomethylated in corpus (< 30% methylation) confirmed the higher methylation of both caput and cauda in the same tracts, homogeneously distributed along all chromosomes (Fig. 3B).

**Fig. 4:**
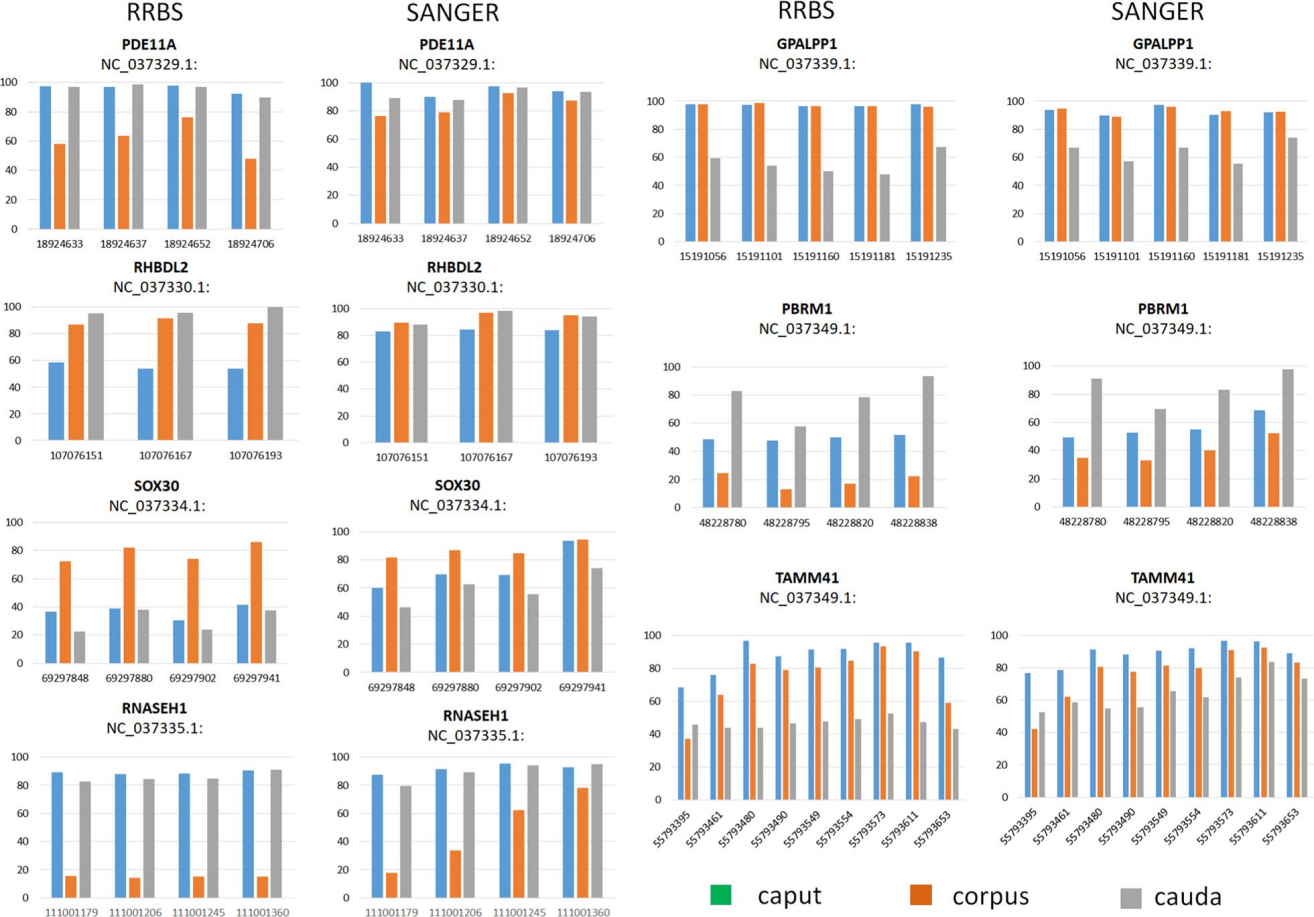
Methylation percentage assessed by bisulfite Sanger Sequencing and by RRBS in cytosines mapping in DMRs in sperm isolated from caput, corpus and cauda. Cytosine positions are reported, together with the gene acronym and the chromosome NCBI RefSeq sequence number

A subset of these cytosines differentially methylated between corpus and the other two regions was chosen for validation by Sanger Sequencing. Results were consistent with methylation profiles obtained by RRBS in DMRs (Fig. 4).

## Discussion

Mammalian spermatozoa undergo structural and biochemical changes during their passage along the epididymis, aimed at achieving optimal fertilizing ability. In most mammals, sperm starts tail beating in the caput and increases motility progressively from the corpus to the cauda [29, 30]. Our results from CASA measurement of bull sperm isolated from different epididymis tracts show a similar trend with motility and velocity increasing from caput to cauda. In addition, we observed that cauda sperm from mature bulls showed significant improvement of motility and sperm kinetics. A previous work in young and adult Swedish Red and White sires showed an age-dependent improvement of sperm motility, CASA measurements, and membrane integrity [31]. Estonian Holstein bulls also showed significant increasing in sperm motility, membrane integrity and acrosome morphology in relation to the age of the sires [32]. On the contrary, young Nellore bulls compared to the older presented superior values for motility, acrosomal membrane integrity and higher mitochondrial potential.

Unexpectedly, cytometric and microscopic measurements showed specific changes and a severely disordered membrane acrosome in sperm isolated from the corpus, suggesting a potential rearrangement of the chromatin structure in this tract.

Analysis of sperm chromatin compaction by Feulgen-like technique showed variation in different epididymis tracts in rabbit [33]. Using the same technique, ram sperm isolated from the corpus of the epididymis showed the greatest decrease in Feulgen-DNA and surface area compared with spermatozoa from the other regions, which indicates a marked restructuration of the chromatin in this region of the epididymis [34]. Immunocytochemical localization of protamine in boar spermatozoa showed a significant decrease in the immunolabelling detected from the corpus, indicating this region as critical for the interaction between DNA and the protamines during the sperm epididymal maturation [35]. Recently, crossbred bulls showed specific alteration of chromatin condensation in different tracts of the epididymis [22]. Our results show specific alteration in CpG methylation in bull sperm isolated from different tracts in two groups of animals differing in aging. Only a small proportion of CpG changed in sperm between young and mature bulls, whereas sperm methylation alteration in different epididymis tracts was more consistent. In bulls, contrasting results were obtained. Sperm CpG methylation changes were observed only in very young bulls, which variation was more related to the early-pubertal stage rather than aging [25]. A study on frozen-thawed semen from Norwegian Red bulls at both 14 and 17 months of age showed similar global sperm DNA methylation, but specific DNA methylation differences were linked to important sperm functions and hormonal pathways [36]. Cryopreserved semen CpG methylation from a single Japanese Black bull collected at five different ages, from 5 to 162 months, showed distinct age-related changes in methylation profiles [37].

Although local moderate changes in CpG methylation were observed to be linked to bull sperm aging, our data depicted a global CpG methylation that clearly separates caput, corpus and cauda sperm isolates. Recently, genome-wide methylation landscape in seven germ cell populations throughout the male reproductive tract in mouse showed DNA methylation variation in caput epididymis but not in other compartments. This variation was susceptible to sperm DNAse treatment and potentially attributed to extracellular DNA [38, 39]. Our data showed only a modest CpG methylation variation between caput and cauda that could be explained by the fact that before isolation sperm was treated with detergents that lysed contaminant somatic cells and potentially eliminate any eventual exogenous DNA. On the contrary, CpG methylation in sperm isolated from the corpus show the highest variation when compared to sperm isolated from the other two epididymis tracts. These results are consistent with sperm phenotype alteration during sperm maturation observed by microscopy investigation.

Corpus epididymis profile is likely to have an intermediate methylation level between caput and cauda, however we observed specific dynamic changes in DNA methylation in that region. Several direct and indirect observations can support our results: (i) our measurements show that epididymis corpus presents significant alterations in variables related to sperm with defect in acrosome integrity, and sperm morphology; (ii) chromatin changing was previously observed to specifically occur in the epididymis corpus [22, 33–35]; (iii) non-coding RNA profiling of bull sperm isolated from testis to ejaculated sperm showed that small RNA sperm content is markedly remodeled as sperm matures along the epididymis, with specific enrichment in ribosomal RNA and also in 20 nt long piRNAs in the epididymis corpus [17], (iv) specific remodeling in histone post-translational modifications was observed in mouse and ram sperm isolated from caput and cauda, which is likely to shape the paternally inherited epigenome and may influence spatial organization of the genome in the early embryo [18]. Unfortunately, this study did not investigate histone modification in the corpus epididymis [18].

All these observations reinforce the conclusion that sperm maturation in the epididymis involves a high epigenetic remodeling that may influence sperm fertility and a correct embryo development.

## Conclusion

In conclusion, according to our data including RRBS, sperm motility and flow cytometry, assessment of bull sperm isolated from different tracts of the epididymis revealed specific variation in sperm quality parameters and epigenetic changes. Corpus epididymis sperm presented significant alterations in variables related to acrosome integrity. CpG methylation alterations in sperm isolated from the corpus epididymal tract are observed, which are likely to refine the sperm epigenome and potentially impact the sperm fertility capacity, as well as the spatial organization of the genome during early embryo development.

## Methods

### Animals

Eight sexually mature Italian Holstein bulls were considered in this experiment. The bulls were classified according to their age of slaughter, in two experimental groups: Young Bulls (YB = n. 4 of 16–19 months at slaughter, B1–B4) and Old Bulls (OB = n. 4 of 45–72 months at slaughter, B5–B8).

### Testicle preparation, sperm recovery and quality assessment

Testicles of each bull were collected at the abattoir within 30 min after slaughtering, and then transported to our laboratories, in a plastic bags in a Styrofoam box containing cold packs at + 4 °C. At the laboratories, epididymides with respective ductus deferens were isolated and cleaned of blood and connective tissue. After that three epididymis tracts well distinct from an anatomical point of view and not adjacent, the caput, the corpus and the cauda tracts with ductus deferens, were surgically separated with the aid of scissors and scalpels, and washed with saline solution.

For each of the tracts different techniques have been used in order to collect the sperm cells, avoiding as much as possible blood, tissue and somatic cells contamination.

After dissecting the caput epididymis from the testicle and the remaining part of the epididymis, the first layer of tunica albuginea has been removed and few tubules were neatly cut with a scalpel; intraluminal fluid was recovered by applying pressure to the proximal tract of the dissected caput epididymis, with a micropipette.

For the corpus tract, particular attention has been paid to try to recover only sperm cells. To overcome this problem the first layer of tunica albuginea has been removed and the microtubules were neatly cut with a scalpel, avoiding the blood microvessels present. Some drops of Tyrode’s albumin–lactate–pyruvate (TALP) medium containing 2 mM penicillamine, 1 mM hypo-taurine, 250 mM adrenaline, 20 mg mL^−1^ heparin, 114 mM NaCl, 3.2 mM KCl, 0.4 mM NaH_2_PO_4_, 10 mM sodium lactate, 25 mM NaHCO_3_, 0.5 mM MgCl_2_ ∙ 6H_2_0, 2.0 mM CaCl_2_ ∙ 2H_2_O, 6 mg mL^−1^ BSA, 5 mg mL^−1^ gentamicin and 0.2 mM sodium pyruvate were dropped on this tract, avoiding leakage from the incised area. After 1 min the semen suspension was collected by using a micropipette. The corpus tract was washed two times.

The intraluminal fluid from the cauda epididymis and ductus deferens was obtained by retrograde flushing as described by Turri et al. [40], cannulating the lumen of the ductus deferens with a blunted 22G needle. Sperm cells were then flushed in a retrograde direction from the ductus deferens through the cauda epididymidis with a syringe loaded with approximately 1 ml of room temperature extender. From each epididymis tract the intraluminal fluid was collected in tubes of 2 ml and split in to two aliquots, one for DNA extraction and one for sperm qualitative evaluation.

### Evaluation of sperm characteristics

Sperm cells of each epididymis tract from each bull was evaluated for total motility and sperm kinetic parameters by Computer-Assisted Semen Analysis (CASA) system and sperm viability, acrosomal status, and DNA integrity by flow cytometer (FCM) immediately after epididymis extraction.

### Computer-assisted semen analysis

Total motility and sperm kinetics parameters were assessed by CASA system (ISAS®v1, Proiser, R + D S.L., Paterna, Spain) combined with a phase contrast microscope (Nikon Optiphot) equipped with a negative phase contrast 10 × objective and integrated warmer stage and connected to a video camera (Proiser 782 M, Proiser R + D). Semen samples from the three epididymis tracts were diluted, respectively, 1:25 for the caput and corpus tracts, and 1:75 for the cauda tract, with Easy Buffer B® (IMV Technologies, L'Aigle, France) and incubated for 15 min at 37 °C. Then 10 µl of diluted semen of each tract were placed on a prewarmed (37 °C) Makler® chamber. During the analysis, the microscope heating stage was maintained at 37 °C. Images were relayed, digitized, and analyzed by the ISAS®v1 software with user-defined settings as follows: frames acquired, 25; frame rate, 20 Hz; minimum particle area 20 µm^2^; maximum particle areas 70 µm^2^; progressivity of the straightness 70%. CASA kinetics parameters were total motility (MOT_TOT, %), progressive motility (PRG, %), curvilinear velocity (VCL, µm/s), straight-line velocity (VSL, µm/s), average path velocity (VAP, µm/s), linearity coefficient (LIN, %, = VSL/VCL × 100), amplitude of lateral head displacement (ALH, µm), straightness coefficient (STR, % = VSL/VAP × 100), wobble coefficient (WOB, % = VAP/VCL × 100), and beat cross-frequency (BCF, Hz).

### Flow cytometry analysis

Measurements were recorded using a Guava EasyCyte™ 5HT microcapillary flow cytometer (Merck KGaA, Darmstadt, Germany; distributed by IMV Technologies, L'Aigle, France) equipped with fluorescent probes excited by a 20-mW argon ion laser (488 nm). Forward-scatter (FSC) vs. side-scatter (SSC) plots were used to separate sperm cells from debris. Non-sperm events were excluded from further analysis. Fluorescence detection was set with three photomultiplier tubes: detector FL-1 (green: 525/30 nm), detector FL-2 (yellow/orange: 586/26 nm), and detector FL-3 (red: 690/50 nm). Calibration was carried out using standard beads (Guava Easy Check Kit, Merck Millipore). A total of 5,000 sperm events per sample for each bull were analyzed at a flow rate of 200 cells/s. Compensation for spectra overlap between fluorochromes was set according to the procedures outlined by Roederer (2000), [41]. Data were acquired and analyzed using CytoSoft and EasyCompDNA software (Merck KGaA, Darmstadt, Germany; distributed by distributed by IMV Technologies, L'Aigle, France), respectively.

### Sperm viability

The LIVE-DEAD® Sperm Viability Kit (Life Technologies Italia, Italy) was used for the analysis of plasma membrane integrity as described by Turri et al. [42]. The kit contained the membrane-permeant nucleic acid stain SYBR®14 and the conventional dead cell stain propidium iodide (PI). Both dyes can be used to label DNA. After staining, live sperm cells with intact cell membranes fluoresced bright green, whereas cells with damaged cell membranes fluoresced red. Aliquots of semen extended with Easy Buffer (2.0 × 10^5^ spermatozoa/ml) were supplemented with SYBR®14 0.1 µM and PI 12 µM (final dilutions) according to manufacturer instructions. After gentle mixing and 10 min incubation at 37 °C in the dark, three replicates per sample were performed. Debris particles were gated out according to the intensity of green and red fluorescence. Three sperm populations were detected on the FL-1/FL-3 dot plot: viable (green), dead (red), and moribund (double-stained) spermatozoa.

### Acrosomal membrane integrity

The fluorescent stains fluorescein isothiocyanate-conjugated peanut agglutinin (FITC-PNA) and propidium iodide (PI) at the appropriate concentrations and 2.0 × 10^5^/ml frozen-thawed sperm cells were added to Easy Buffer B® (IMV Technologies, L'Aigle, France). FITC-PNA labeled reacted acrosomes (green), whereas PI-stained dead sperm cells (red). Samples were incubated for 45 min at 37 °C in the dark. The protocol supplied by IMV Technologies was followed; the procedure, based on a confidentiality agreement, cannot be disclosed in detail. Three replicates were performed per sample. Compensations were set according to the procedures outlined by Roederer [41]. After gating out non-DNA containing particles, four sperm populations were detected on the FL-1/FL-3 dot plot: live and dead spermatozoa, with intact and ruptured acrosomes.

### Sperm chromatin structure assay

The sperm chromatin structure assay was assessed by using acridine orange, a planar molecule that intercalates into double-stranded DNA but stacks on single-stranded DNA causing a metachromatic shift from green (double-stranded DNA) to red fluorescence (single-stranded DNA), when exposed to the 488 nm laser light of the flow cytometer. The technique is based on the susceptibility of sperm DNA to acid-induced denaturation as low pH treatment causes partial DNA denaturation in sperm with altered chromatin structure. The assessments were performed using the sperm chromatin structure assay [43]: 3.0 × 10^5^ cells were diluted in 200 µl of TNE buffer (0.01 M Tris–HCl, 0.15 M NaCl, 1 mM EDTA, pH 7.4) and added to 400 µl of an acidic solution (Triton X-100 0.1%, 0.15 M NaCl, 0.08 N HCl; pH 1.2). After 30 s, cells were stained with 1.2 ml of acridine orange solution (6 µg/ml in 0.1 M citric acid, 0.2 M Na2HPO4, 1 mM EDTA, 0.15 M NaCl; pH 6). Samples were protected from light and incubated at room temperature for 2.5 min; after that, two replicates per sample were acquired and analyzed using, respectively, CytoSoft and EasyCompDNA software (Merck KGaA, Darmstadt, Germany; distributed by IMV Technologies, L'Aigle, France) and expressed as Alpha-T, which is indicative of the shift from green to red fluorescence, is expressed as the ratio of red to total fluorescence intensity [red/(red + green)], and quantifies the degree of abnormal chromatin structure with an increased susceptibility to acid-induced denaturation; standard deviation of Alpha-T (ATSD), which shows the extent of abnormality in the chromatin structure within a population; DNA fragmentation index (%DFI), which indicates the percentage of sperm with fragmented DNA; a percentage of sperm with high green fluorescence (%HG), which is representative of the percentage of immature cells with reduced nuclear condensation (incomplete histone–protamine exchange); the High DNA stability (HDS) that represents the percentage of immature sperm. The FCM instrument was AO-saturated prior to analysis by running the AO equilibration solution.

### Microscope assessment of sperm acrosome membrane

Sperm acrosomal status was also evaluated by fluorescence microscope, in order to visually detect structural modifications in sperm head membranes trough the three different epididymis tracts.

Ten µl of semen samples collected from each tracts were spread across the glass slide surface and allowed to air dry. Slides were stained with fluorescein isothiocyanate-conjugated peanut agglutinin (FITC-PNA) (Life Technologies) following the protocol of Roth et al. [44] with some modifications. Briefly, an aliquot of stock FITC-PNA (1 mg/ml) was thawed and added to 900 µl PBS for a final concentration of 100 µg/ml. Twenty µl of this working solution were spread on the slides, subsequently incubated at 4 °C for 15 min in the dark. After incubation, slides were washed in PBS at 4 °C and were allowed to air dry in the dark. Then slides were permanently sealed with Eukitt mounting medium (O. Kindler GmbH, Freiburg, Germany) and topped with a coverslip. Two separate smears were prepared for each epididymidis. Two hundred cells for smear were assessed by using a phase contrast microscope (Leica DM-LB 30 FLUO; Leica Microsystems Imaging Solutions Ltd, Cambridge, UK) with a × 100 oil immersion objective.

The staining patterns of the acrosomes were classified into three categories identified by Harayama et al. [45]: slightly disordered acrosome, severely disordered acrosome; intact acrosome with normal membrane.

### DNA extraction

DNA extraction was performed on sperm isolated from the different epididymal tracts following a previously published method, with some modifications [17]. Briefly, the sperm epididymal fractions were centrifuged at 2500 *g* × 5' at 4 °C to recover the cellular pellet, that was washed once in PBS buffer and centrifuged at 2500 *g* × 5' at 4 °C. Pellet was resuspended in buffer containing 0.1% SDS and 0.5% Triton X-100 for 10’ at 4 °C to lysate somatic cells, and centrifuged 2500 *g* × 5' at 4 °C. Pellet was washed once with PBS and finally centrifuged 7000 *g* 4' at 4 °C to recover the sperm pellet, that was microscopically inspected for absence of contamination by somatic cells and stored at − 80 °C. DNA was extracted by NucleoSpin® Tissue (Macherey–Nagel) with an incubation with proteinase K and following manufacturer instruction. Isolated DNA was stored at − 20 °C.

### Reduced representation library preparation and sequencing

About 1 μg of genomic DNA from each sperm pellet collected (caput, corpus, cauda isolates from eight animals, *n* = 24) was digested with MspI (New England Biolabs, Ipswich, MA, USA) by overnight incubation at 37 °C, following the manufacturer’s instructions. Libraries were prepared with TruSeq DNA PCR-Free Library Preparation Kit (Illumina, San Diego, CA, USA), with some modifications. After adapter ligation, samples were bisulfite treated with the EpiTect Bisulfite Kit (Qiagen, Venlo, The Netherlands) and finally PCR amplified with KAPA HiFi Uracil + kit (Kapa Biosystems, Potters Bar, UK). RRBS libraries were sequenced on an Illumina Hiseq X (San Diego, CA, USA) to generate 150-base paired-end reads.

### Bioinformatics analysis

Preliminary quality control of raw reads was carried out with FastQC (http://www.bioinformatics.babraham.ac.uk/projects/fastqc/). Illumina raw sequences were then filtered with TrimGalore (http://www.bioinformatics.babraham.ac.uk/projects/trim_galore/) to remove adapters and low quality bases at the ends of sequence, using RRBS-specific parameters. Data are available in the Sequence Reads Archive (SRA), (Accession Number PRJNA874884). The Bismark software v.0.17.0 (https://www.bioinformatics.babraham.ac.uk/projects/bismark/) was used to align each read to a bisulfite-converted *Bos taurus* reference genome (ARS-UCD1.2: GCF_002263795.1), and methylation calls were recovered using the Bismark methylation_extractor function. The Seqmonk software (version 1.48.0) was used for visualization and analysis of the Bismark output (http://www.bioinformatics. babraham.ac.uk/projects/seqmonk/).

Only positions with at least 10X cytosines were recorded in all samples and used for RRBS analysis. Methylated regions (MRs) were detected by dividing the genome in 400-bp tiles containing at least three mutually covered Cs in the CpG context per position tiles and analyzing average methylation in a sliding window of 200 bp. MRs were considered if quantified in all 24 samples. The overall MRs distribution in all the samples was assessed by principal component analysis (PCA) and hierarchical clustering using Genesis Software [46]. Differentially methylated regions (DMRs) between caput–corpus, caput–cauda and corpus–cauda were calculated using the Edge-R statistical package (Bioconductor, https://bioconductor.org/packages/release/bioc/html/edgeR.html), (FDR < 0.05, absolute cutoff of 10%) [47].

### Bisulfite Sanger sequencing PCR

A subset of positions showing highest CpG methylation variation across the three epididymis tracts were chosen for validation using bisulfite Sanger sequencing PCR. Primers were designed using MethPrimer [48] and are listed in additional file 6. DNA isolated from 8 animals for each epididymis tract was pooled and treated with bisulfite with the EpiTect Bisulfite Kit (Qiagen, Venlo, The Netherlands). PCR products for each gene were amplified with KAPA HiFi Uracil + kit (Kapa Biosystems, Potters Bar, UK) and specific primers with the following cycling conditions: 5’ at 95 °C, followed by 32 cycles of 20″ at 98 °C, 20″ at 58 °C and 60″ at 72 °C, and finally 7’ at 72 °C. Region-specific CpG methylation level was obtained using the automated R tool ABSP [49].

### Statistical analysis

Data obtained from CASA and FCM measurements were analyzed using the SASTM package v 9.4 (SAS Institute Inc., Cary, NC, USA). In particular, the PROC MIXED procedure was used to perform analysis of covariance of in vitro sperm parameters, in the different epididymal tracts. The mixed model included the fixed effects of bulls age and of epididymis tracts, categorized as young bulls (16–19 months) and adult bulls (45–72 months). The bull effect was considered as random.

## Supplementary Information

**Additional file 1.** Quality parameters for sperm isolated from caput, corpus, cauda epididymis tracts between young and old bulls.

**Additional file 2.** Mapping and methylation statistics.

**Additional file 3.** Differentially methylated regions identified for the comparison between young and old bulls.

**Additional file 4.** Total methylated regions and differentially methylated regions identified for the three comparison

**Additional file 5.** Venny Diagram of 8,945 DMRs shared between caput vs corpus, corpus vs cauda.

**Additional file 6.** List of primers used for Bisulfite Sanger Sequencing validation.

## Data Availability

All sequence data are deposited at the NCBI Sequence Read Archive (SRA) (https://www.ncbi.nlm.nih.gov/sra) (Accession Number PRJNA874884).

## Abbreviations

RRBS: Reduced representation bisulfite sequencing
CpG: Cytosines in CpG dinucleotides
MRs: Methylated regions
DMRs: Differentially methylated regions
DMGs: Differentially methylated genes
GO: Gene Ontology
FDR: False discovery rate
FCM: Flow cytometry technique
SCSA: Sperm chromatin structure assay
MOT TOT: Total motility
STAT: Immotile cells
PROG: Cells progressive motility
VSL: Straight-line velocity
VCL: Curvilinear velocity
VAP: Average path velocity
LIN: Linear coefficient
STR: Straightness coefficient
WOB: Wobble coefficient
ALH: Amplitude of lateral head displacement
BCF: Beat cross-frequency
V: Viable sperm
D: Death sperm
VI: Viable with intact acrosome
VR: Viable with disrupted acrosome
DI: Death with intact acrosome
DR: Death with disrupted acrosome
Alpha-T: Red/(red + green) fluorescence intensity
ATSD: Alpha-T standard deviation
DFI: Fragmented DNA sperm
HG: High green fluorescence sperm
HDS: Proportion of immature spermatozoa lacking the normal exchange of histone for protamine-complexed DNA
