# Supplementary material for: CpG DNA methylation changes during epididymal sperm maturation in bulls
Source: Epigenetics Chromatin. 2023 May 30;16:20. doi: 10.1186/s13072-023-00495-6 (PMC10228035; doi:10.1186/s13072-023-00495-6)
Supplement: Supplementary file 1 — Additional file 1. Quality parameters for sperm isolated from caput, corpus, cauda epididymis tracts between young and old bulls. [file 13072_2023_495_MOESM1_ESM.docx]

|  | **caput** | | **corpus** | | **cauda** | |  |
| --- | --- | --- | --- | --- | --- | --- | --- |
| **Parameters** | **young** | **old** | **young** | **old** | **young** | **old** | **Significativity** |
| MOT | 70,27±8,81 | 53,25±8,82 | 76,70±8,81 | 63,92±8,81 | 75,47±8,81 | 73,02±8,81 | NS |
| STAT | 29,72±8,81 | 46,75±8,82 | 23,30±8,81 | 36,07±8,81 | 24,52±8,81 | 26,97±8,81 | NS |
| PROG | 16,32±3,24 | 13,85±3,24 | 19,40±3,24 | 19,60±3,24 | **32,70±3,24 a** | **47,17±3,24 b** | <0.001 |
| NOPROG | 53,95±7,52 | 39,27±7,53 | 57,35±7,52 | 44,37±7,52 | 42,77±7,52 | 25,82±7,52 | NS |
| VCL | 93,94±5,28 | 88.03±5,28 | 96,22±5,28 | 101,92±5,28 | **107,85±5,28a** | **135,21±5,28b** | <0.001 |
| VSL | 8,66±9,38 | 13,84±5,46 | 9,88±9,38 | 20,64±5,44 | **34,82±9,38 a** | **70,35±5,46 b** | <0.0001 |
| VAP | 27,86±8,36 | 28,33±4,98 | 28,87±8,36 | 37,22±4,98 | **51,16±8,36 a** | **83,95±4,98 b** | <0.001 |
| LIN | 3,13±7,36 | 13,80±3,98 | **3,94±7,36 a** | **17,41±3,98 b** | **24,60±7,36b A** | **48,87±3,98 B** | <0.0001 |
| STR | 53,10±3,63 | 57,73±3,63 | 55,14±3,63 | 59,52±3,63 | 72,25±3,63 | 85,05±3,63 | NS |
| WOB | 19,81±5,67 | 28,66±3,02 | **19,92±5,67 a** | **32,37±3,02 b** | **36,32±5,67 A** | **57,61±3,02 B** | <0.0001 |
| ALH | 3,67±0,23 | 3,38±0,23 | **3,85±0,23 a** | **4,01±0,23 b** | **3,73±0,23 A** | **4,21±0,23 B** | <0.0001 |
| BCF | 3,14±0,92 | 3,44±0,49 | **2,59±0,92 a** | **5,10±0,49 b** | **4,20±0,92 A** | **7,00±0,49 B** | <0.001 |
| V | **36,62±9,34 a** | **54,23±5,03 b** | 32,10±9,34 | 41,93±5,03 | **41,43±9,34 A** | **57,14±5,03 B** | <0.05 |
| M | **64,20±9,37a** | **46,08±5,04b** | 69,35±9,37 | 58,38±5,04 | **59,47±9,37A** | **43,17±5,04B** | <0.01 |
| VI | **34,94±9,37 a** | **52,74±5,04 b** | 28,72±9,37 | 39,21±5,04 | **38,93±9,37 A** | **55,07±5,04 B** | <0.05 |
| VR | 0,85±0,48 | 1,17±0,48 | 2,54±0,48 | 2,40±0,48 | 1,67±0,48 | 1,75±0,48 | NS |
| MR | 9,37±2,47 | 6,20±1,37 | 13,69±2,47 | 9,64±1,37 | 11,03±2,47 | 8,85±1,37 | NS |
| MI | **54,22±7,53 a** | **39,65±4,05 b** | 55,05±7,53 | 48,50±4,05 | **47,83±7,53 A** | **34,08±4,05 B** | <0.05 |
| ALPHAT | 0.4092±0.0036 | 0.4115±0.0029 | 0.4080±0.0036 | 0.4064±0.0028 | 0.4035±0.0036 | 0.4036±0.0032 | NS |
| SD | **0.0005±0.0045 a** | **0.0049±0.0024 b** | 0.0034±0.0045 | 0.0099±0.0024 | **0.0012 ±0.0045 A** | **0.0092 ±0.0026 B** | <0.05 |
| DFI | 0.8975±0.3308 | 1.1100±0.3308 | **1.1475±0.3308 a** | **2.1300±0.3308 b** | 1.5425±0.3308 | 1.5317±0.3308 | <0.05 |
| HG | 1,6175±0,6297 | 1.4375±0,6297 | 1,6775±0,6297 | 2,5538±0,6297 | 0,8725±0,6297 | 0,4500±0,7271 | NS |
| HDS | 3,3600±0,6658 | 3.3338±0,6658 | 3,5725±0,6658 | 3,4913±0,6658 | 3,3525±0,6658 | 3,2583±0,7688 | NS |

MOT TOT, total motility; STAT, immotile cells; PROG, cells progressive motility; VSL, straight-line velocity; VCL,curvilinear velocity; VAP, average path velocity; LIN, linear coefficient; STR, straightness coefficient; WOB, wobble coefficient; ALH, amplitude of lateral head displacement;BCF, beat cross-frequency; V, viable sperm; m, death sperm; VI, viable with intact acrosome; VR,viable with disrupted acrosome; MI, death with intact acrosome; VR death with disrupted acrosome; ALPHA-T, red/(red+ green) fluorescence intensity;ATSD, Alpha-T standard deviation; %DFI, fragmented DNA sperm; %HG, high green fluorescence sperm.HDS: proportion of immature spermatozoa lacking the normal exchange of histone for protamine-complexed DNA

**Additional file 1.** Quality parameters for sperm isolated from caput, corpus, cauda epididymis tracts between young and old bulls.
