# Supplementary material for: CpG DNA methylation changes during epididymal sperm maturation in bulls
Source: Epigenetics Chromatin. 2023 May 30;16:20. doi: 10.1186/s13072-023-00495-6 (PMC10228035; doi:10.1186/s13072-023-00495-6)
Supplement: Supplementary file 2 — Additional file 2. Mapping and methylation statistics. [file 13072_2023_495_MOESM2_ESM.docx]

**Additional file 2.** Mapping and methylation statistics. B1-B8=Bulls. Caput-corpus-cauda=epididymis tracts

| **Sample** | **Total Reads** | **Mapped Read** | **% Mapped Reads** | **Methylated CpGs** | **Unmethylated CpGs** | **% Bisulfite Conversion** | **% Methylated CpGs** |
| --- | --- | --- | --- | --- | --- | --- | --- |
| B1_caput | 21533708 | 12574455 | 58.4 | 35445119 | 13523852 | 97.9 | 72.4 |
| B1_corpus | 24397235 | 13242666 | 54.3 | 37028622 | 15138267 | 98.2 | 71.0 |
| B1_cauda | 70017540 | 47688432 | 68.1 | 76182052 | 27326268 | 98.3 | 73.6 |
| B2_caput | 21794294 | 12558520 | 57.6 | 37310845 | 14125545 | 98.6 | 72.5 |
| B2_corpus | 19435478 | 11147325 | 57.4 | 32897152 | 12590787 | 99.7 | 72.3 |
| B2_cauda | 44313276 | 27990497 | 63.2 | 67687871 | 23969456 | 99.0 | 73.8 |
| B3_caput | 19126337 | 11203425 | 58.6 | 33701962 | 13089523 | 99.0 | 72.0 |
| B3_corpus | 19235771 | 10782181 | 56.1 | 37148689 | 14508928 | 98.9 | 71.9 |
| B3_cauda | 34615589 | 21499820 | 62.1 | 43138996 | 20123283 | 97.8 | 68.2 |
| B4_caput | 30020478 | 18491398 | 61.6 | 50378821 | 19217913 | 97.6 | 72.4 |
| B4_corpus | 22624577 | 12896759 | 57.0 | 41384015 | 16811800 | 98.7 | 71.1 |
| B4_cauda | 19447514 | 11614829 | 59.7 | 29287251 | 11248910 | 98.8 | 72.2 |
| B5_caput | 34893739 | 23342239 | 66.9 | 50150885 | 16451189 | 97.3 | 75.3 |
| B5_corpus | 21770731 | 12724250 | 58.4 | 38970428 | 16294486 | 98.8 | 70.5 |
| B5_cauda | 51785084 | 36068982 | 69.7 | 58298241 | 21983486 | 99.0 | 72.6 |
| B6_caput | 21784187 | 12751428 | 58.5 | 39093710 | 14299697 | 98.4 | 73.2 |
| B6_corpus | 21495058 | 11964086 | 55.7 | 36354352 | 15330698 | 98.5 | 70.3 |
| B6_cauda | 128368477 | 94139014 | 73.3 | 101466598 | 48503168 | 98.9 | 67.7 |
| B7_caput | 24925369 | 13207436 | 53.0 | 39711651 | 14276740 | 97.6 | 73.6 |
| B7_corpus | 26859926 | 15170997 | 56.5 | 47755569 | 20542235 | 98.5 | 69.9 |
| B7_cauda | 98203309 | 62066079 | 63.2 | 86204548 | 36122315 | 97.9 | 70.5 |
| B8_caput | 20694863 | 9404625 | 45.4 | 34351222 | 12187454 | 98.6 | 73.8 |
| B8_corpus | 12640123 | 6930935 | 54.8 | 23919393 | 9503268 | 99.8 | 71.6 |
| B8_cauda | 104839092 | 71433349 | 68.1 | 102229455 | 41947648 | 98.1 | 70.9 |
