# Supplementary figures and images for: CpG DNA methylation changes during epididymal sperm maturation in bulls
Source: Epigenetics Chromatin. 2023 May 30;16:20. doi: 10.1186/s13072-023-00495-6 (PMC10228035; doi:10.1186/s13072-023-00495-6)

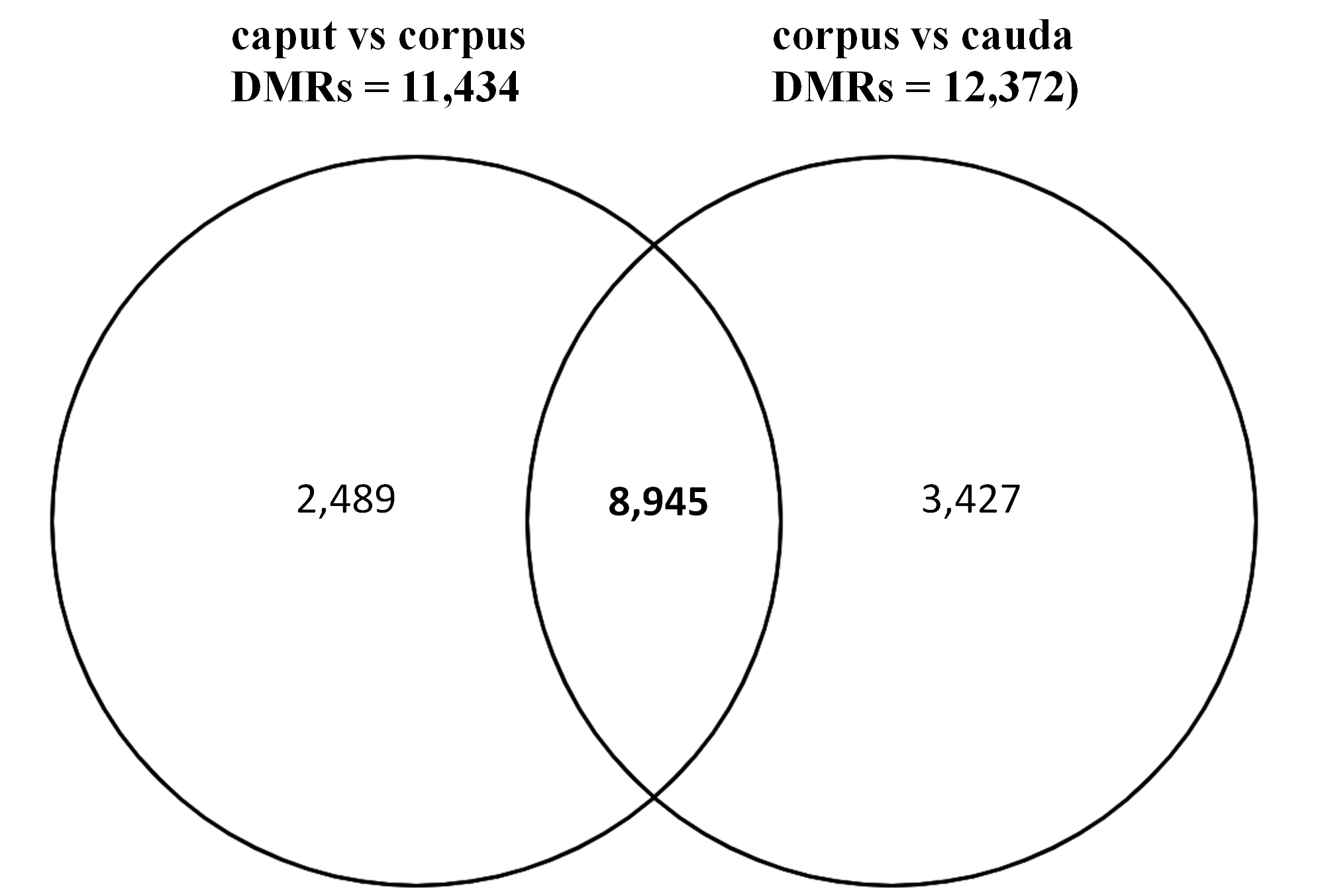


**Additional File 5.** Venny Diagram of 8,945 DMRs shared between caput vs corpus (DMRs = 11,434), corpus vs cauda (DMRs = 12,372).

Supplement: Supplementary file 5 — Additional file 5. Venny Diagram of 8,945 DMRs shared between caput vs corpus, corpus vs cauda. [file 13072_2023_495_MOESM5_ESM.docx]
