# Supplementary material for: CpG DNA methylation changes during epididymal sperm maturation in bulls
Source: Epigenetics Chromatin. 2023 May 30;16:20. doi: 10.1186/s13072-023-00495-6 (PMC10228035; doi:10.1186/s13072-023-00495-6)
Supplement: Supplementary file 6 — Additional file 6. List of primers used for Bisulfite Sanger Sequencing validation. [file 13072_2023_495_MOESM6_ESM.docx]

| **GENE** | **Position** | **Forward Primer** | **Reverse Primer** | **Size (bp)** |
| --- | --- | --- | --- | --- |
| PDE11A | NC_037329.1:18924501-18925100 | AGAAGGAGGATATTTTTGGGATAGA | ATTAACAAAACCTCAAACAACTCAC | 344 |
| RHBDL2 | NC_037330.1:107075901-107076500 | AGAGATAGTAAGAGAGTTTTTTTTAATGTT | ACACTTCACTTCTATAATCTTCCTCC | 390 |
| SOX30 | NC_037334.1:69297501-69298100 | TATTGATTTTTAAGGGGTAAAGTTT | ATATCCAAATAAAAAAATAATATAAAATCA | 348 |
| RNASEH1 | NC_037335.1:111001101-111001700 | TTTTAGGGGATTTTTTTAGTTTAGGA | TAATTCAATAAACAAATTTAAACAAACTCA | 425 |
| GPALPP1 | NC_037339.1:15190850-15191506 | TTTTTGAGGAAATATTGTATTTGTTTATTT | AACCTTCCCCTCTACCTTATTAAAAC | 363 |
| PBRM1 | NC_037349.1:48228501-48229100 | TTTTGGAGTTAATGGATTTGAAAATA | CAAACAAAAAACTTAAAACTCTATAAATTT | 365 |
| TAMM41 | NC_037349.1:55793301-55793900 | GAGGAGATTTGGGTAAATAGTTTTTT | ACCATTACCATAACTATATAACTTTAAACA | 428 |

**Additional File 6.** List of primers used for Bisulfite Sanger Sequencing validation.
